# Developing vaccines against epidemic-prone emerging infectious diseases

**DOI:** 10.1007/s00103-019-03061-2

**Published:** 2019-11-27

**Authors:** Valentina Bernasconi, Paul A. Kristiansen, Mike Whelan, Raúl Gómez Román, Alison Bettis, Solomon Abebe Yimer, Céline Gurry, Svein R. Andersen, Debra Yeskey, Henshaw Mandi, Arun Kumar, Johan Holst, Carolyn Clark, Jakob P. Cramer, John-Arne Røttingen, Richard Hatchett, Melanie Saville, Gunnstein Norheim

**Affiliations:** 1 Coalition for Epidemic Preparedness Innovation (CEPI), Marcus Thranes Gate 2, 0473 Oslo, Norway; 2 Coalition for Epidemic Preparedness Innovation (CEPI), NW1 2BE London, UK; 3 Coalition for Epidemic Preparedness Innovation (CEPI), Washington, DC USA; 4 Research Council of Norway, Lysaker, Norway

**Keywords:** Nipah, MERS-CoV, Chikungunya, Rift Valley fever, CEPI, Nipah, MERS-CoV, Chikungunya, Rifttalfieber, Coalition for Epidemic Preparedness Innovation

## Abstract

Today’s world is characterized by increasing population density, human mobility, urbanization, and climate and ecological change. This global dynamic has various effects, including the increased appearance of emerging infectious diseases (EIDs), which pose a growing threat to global health security.

Outbreaks of EIDs, like the 2013–2016 Ebola outbreak in West Africa or the current Ebola outbreak in Democratic Republic of the Congo (DRC), have not only put populations in low- and middle-income countries (LMIC) at risk in terms of morbidity and mortality, but they also have had a significant impact on economic growth in affected regions and beyond.

The Coalition for Epidemic Preparedness Innovation (CEPI) is an innovative global partnership between public, private, philanthropic, and civil society organizations that was launched as the result of a consensus that a coordinated, international, and intergovernmental plan was needed to develop and deploy new vaccines to prevent future epidemics.

CEPI is focusing on supporting candidate vaccines against the World Health Organization (WHO) Blueprint priority pathogens MERS-CoV, Nipah virus, Lassa fever virus, and Rift Valley fever virus, as well as Chikungunya virus, which is on the WHO watch list. The current vaccine portfolio contains a wide variety of technologies, ranging across recombinant viral vectors, nucleic acids, and recombinant proteins. To support and accelerate vaccine development, CEPI will also support science projects related to the development of biological standards and assays, animal models, epidemiological studies, and diagnostics, as well as build capacities for future clinical trials in risk-prone contexts.

## Zusammenfassung

Eine globale Dynamik geprägt von zunehmender Bevölkerungsdichte, menschlicher Mobilität, Urbanisierung sowie Veränderung klimatischer und ökologischer Bedingungen lässt die zunehmende Ausbreitung von Infektionserregern wie auch das Auftreten neuer Infektionskrankheiten erwarten (EID-emerging infectious diseases). Ausbrüche von EID, wie der Ebola-Ausbruch in Westafrika 2013–2016 oder der aktuelle Ebola-Ausbruch in der Demokratischen Republik Kongo seit August 2018, stellen nicht nur eine gesundheitliche Bedrohung für die Bevölkerungen dar, sie haben auch erhebliche Auswirkungen auf das Wirtschaftswachstum in den betroffenen Regionen und darüber hinaus, und stellen somit eine wachsende Bedrohung für die globale Gesundheit dar. Die Coalition for Epidemic Preparedness Innovation (CEPI) ist eine innovative globale Partnerschaft zwischen öffentlichen, privaten, philanthropischen und zivilgesellschaftlichen Organisationen. Ihre Gründung basiert auf den Konsensus, dass ein koordinierter, internationaler und zwischenstaatlicher Plan erforderlich ist, um neue Impfstoffe zu entwickeln, einzusetzen und so zukünftige Epidemien zu verhindern. CEPI konzentriert sich auf die Unterstützung von Impfstoffkandidaten gegen die in der Blueprint-Liste der Weltgesundheitsorganisation angeführten Erreger. Das aktuelle Portfolio umfasst eine Vielzahl von Technologien, die von rekombinanten viralen Vektoren über Nukleinsäuren bis hin zu rekombinanten Proteinen reichen. Um die Entwicklung von Impfstoffen zu unterstützen und zu beschleunigen, konzentriert sich CEPI auch auf wissenschaftliche Projekte im Zusammenhang mit der Entwicklung biologischer Standards und Assays, Tiermodellen, epidemiologischen Studien und Diagnostika sowie auf den Aufbau von Kapazitäten für zukünftige klinische Studien in entsprechenden Risikogebieten.

## Background

Global trends, including increasing population density, urbanization, human mobility, and climate and ecological change, are leading to emerging infectious diseases (EIDs) that pose a growing threat to global health security [1]. If a highly contagious and lethal airborne pathogen with the characteristics of the 1918 pandemic influenza were to emerge today, it is estimated that nearly 33 million people might die in just 6 months worldwide [2].

The costs of EIDs are enormous, both in terms of lives lost and economic burden. A report prepared by the U.S. National Academy of Sciences in 2016 estimated that over 10 years the global costs of epidemics could amount to US$600 billion, or 0.7% of global income. The cost of a severe pandemic like the 1918 influenza pandemic could total as much as 5% of global gross domestic product (GDP). Even when the health impact of an outbreak is relatively limited, its economic consequences can quickly become magnified [3]. Liberia, for example, saw GDP growth decline 8% from 2013 to 2014 during the Ebola outbreak in West Africa, even as the country’s overall mortality rate fell over the same period [4].

From the beginning of the 21st century to the present, the world has experienced several outbreaks of EIDs, with considerable public health concerns: Severe acute respiratory syndrome-related coronavirus (SARS-CoV) in 2003–2004, H1N1 “swine flu” in 2009, Middle East respiratory syndrome coronavirus (MERS-CoV) since 2012, Ebola virus in 2013–2016 and from 2018 onward, and Zika virus in 2015–16 are some examples [5–9]. In each instance, it was not possible to predict the time, location, or identity of the causative pathogen beforehand [10].

Vaccination is a critical tool in the response to these unpredictable outbreaks as well as, eventually, in their prevention. However, the complete process for bringing a vaccine from the research laboratory to the population is long, complex, and expensive, typically requiring a capital investment of US$500 million to US$1 billion over a period of 10 years [11].

### The importance of vaccines and challenges in their development

Vaccination has been described as one of the most successful public health interventions to date [12]. Our modern vaccinology era started when Edward Jenner, an English general practitioner, conducted the first scientific investigation on smallpox prevention in 1796 [13]. Since then, vaccinations have reduced disease, disability, and death from a variety of infectious diseases all over the world [12]. Despite this success, there is still a great need for new vaccines that can prevent and reduce the impact of outbreaks of both endemic infectious diseases and emerging infectious diseases [14]. In the case of EIDs, this is especially challenging due to the fact that the identity of the pathogen responsible for the disease, as well as the time and location of the next outbreak, cannot be accurately predicted using current means [15, 16].

Historically, vaccine development has been a long, risky, and costly endeavor. Planning vaccination against EIDs is especially challenging: The potential market for vaccines against these diseases is limited, and testing such vaccines is difficult [17]. Several bottlenecks have been identified in the development of vaccines against EIDs [18].

The first limiting factor is related to the preclinical discovery: understanding the pathogenesis mechanism, developing the appropriate animal-challenge models, and being able to screen, test, and generate the proof of concept (PoC) for new antigens and delivery platforms is not trivial [18]. Moreover, preclinical development is a complex, multistep, and time-consuming process. This represents the second bottleneck in the vaccine development process and involves selection/screening of appropriate antigens and verification of efficacy in the animal models. This is followed by process development to ensure that a scalable, robust, and good manufacturing practice (GMP)-compliant process is established. Material generated at the end of preclinical development can be used for animal toxicology studies and forms the basis of a clinical trial application [18].

The traditional clinical trial phases require significant investment and resources to be executed, and the lengthy nature of the process could itself be described as a bottleneck. Many EIDs are prone to sporadic outbreaks in which morbidity and mortality are high, and it is sometimes not possible to conduct traditional phase III efficacy trials due to ethical considerations and the scale and unpredictable nature of EID outbreaks [18]. To meet the unique challenges of vaccine development for EIDs, an innovative, efficient global system of vaccine research and development (R&D) for EIDs is needed [10].

### The creation of CEPI

After the devastating West African Ebola epidemic in 2013–16, which alone claimed the lives of more than 11,000 people and had a comprehensive economic and social burden estimated at over US$53 billion (or more than $1.8 million per case), the global need for an organization that could finance and coordinate the development of vaccines against EIDs was recognized [19]. In 2014, although there was no licensed Ebola vaccine available, approximately 15 different vaccines were in preclinical development, including DNA vaccines, virus-like particles (VLPs), and viral vector-based vaccines [20]. It took a year to initiate field trials of the first Ebola vaccines, many of which had been under development for more than a decade. It became evident that an improved system for the development of vaccines against known and unknown epidemic threats was needed [21].

The early ideas for establishing what became the Coalition for Epidemic Preparedness Innovations (CEPI) were consolidated at the World Economic Forum Annual Meeting in Davos in January 2016, and CEPI was launched 1 year later to facilitate and fund coordinated, international, and intergovernmental planning to develop and deploy new vaccines to prevent and reduce the impact of EID epidemics. The Coalition is an innovative global partnership between public, private, philanthropic, and civil society organizations, and its mission is to stimulate and accelerate the development of vaccines against EIDs and enable access to these vaccines for people affected by outbreaks [22].

It was founded by the governments of Norway and India, the Bill & Melinda Gates Foundation, the Wellcome Trust, and the World Economic Forum. From 2017 CEPI has secured approximately US$820 million of direct and aligned investments toward its US$1 billion funding target, including multiyear funding from Norway, Germany, Japan, Canada, Australia, the European Commission, the Bill & Melinda Gates Foundation, and the Wellcome Trust. It has also received single-year investments from the governments of Belgium and the UK (Table 1; [22]).

**Table 1:** Coalition for Epidemic Preparedness Innovations investors (as of November 2019). Conversion rates as per November 2019

Investor	Investment amount (local currency)	Investment amount (US$ million)	Type of investment
Norway	Norwegian krone 1.6 billion	191.8	Multiyear
Japan	US$125 million	125	Multiyear
Germany	€90 million	102.4	Multiyear
Wellcome Trust	US$100.4 million	100.4	Multiyear
Bill & Melinda Gates Foundation	US$100 million	100	Multiyear
European Commission	€80 million	89.6	Multiyear
United Kingdom	£10 million	12.6	Single year
Canada	Canadian $14 million	10.7	Multiyear
Australia	Australian $6.5 million	4.9	Multiyear
Belgium	€0.5 million	0.6	Single year

### Filling a critical gap in the vaccine “ecosystem”

**Fig. 1:**
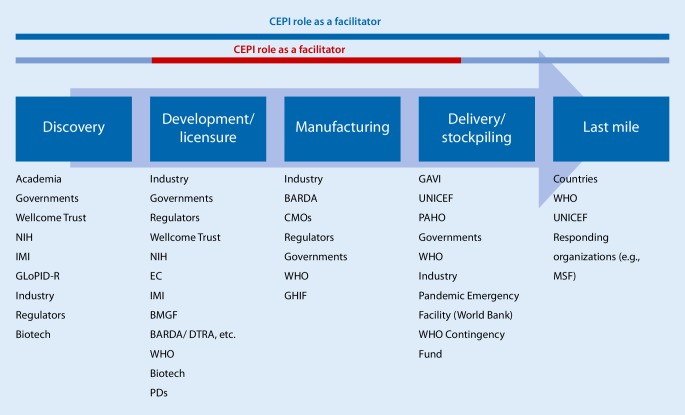
The role of the Coalition for Epidemic Preparedness Innovations (CEPI) within the vaccine development pipeline; CEPI is funding projects from phase I trials to the development of a stockpile and has a role as facilitator in the vaccine development process from discovery to the delivery and stockpiling of new vaccines. (*NIH* National Institutes of Health; *IMI* Innovative Medicines Initiative; *GloPID‑R* Global Research Collaboration for Infectious Disease Preparedness; *EC* European Commission; *BMGF* Bill & Melinda Gates Foundation; *BARDA* Biomedical Advanced Research and Development Authority; *DTRA* Defense Threat Reduction Agency; *WHO* World Health Organization; *PDs* product developers; *CMOs* contract manufacturing organizations;* GHIF* Global Health Investment Fund; *GAVI* Global Alliance for Vaccines and Immunization; *UNICEF* United Nations International Children’s Emergency Fund; *PAHO* Pan American Health Organization; *MSF* Médecins Sans Frontières)

Many organizations operate within the end-to-end space of vaccine funding and R&D implementation. However, several critical gaps have been identified, which CEPI is designed to fill (Fig. 1). The R&D is complex, lengthy and expensive; the potential market for such vaccines against EIDs is very limited; and testing of such vaccines is difficult [17].

CEPI is designed to advance vaccines against known threats through PoC and safety testing in humans and establishing investigational stockpiles to be used emergently at the beginning of an epidemic under a clinical trial regimen. It also funds new and innovative platform technologies that carry the potential to accelerate the development and manufacturing of vaccines against previously unknown pathogens. Moreover, CEPI coordinates activities to improve the collective response to epidemics, strengthening capacity in countries at risk and advancing the regulatory science that governs product development.

CEPI has three strategic objectives: *preparedness, response*, and *sustainability*, and it aims to advance safe and effective vaccines against EIDs; accelerate the research, development, and use of vaccines during outbreaks; and create durable and equitable solutions for outbreak response capacity [22]. It offers a unique opportunity for investors to lead on global health security and, in partnership with other governments and international organizations, invest in solutions that aim to protect some of the most vulnerable people in the world while helping prevent the spread of epidemics [22].

## The Coalition for Epidemic Preparedness Innovations portfolio

### WHO R&D Blueprint

The World Health Organization (WHO) developed a list of diseases and pathogens to be prioritized for research and development under the WHO R&D Blueprint for emerging infections. Diseases were prioritized on the basis that they pose a public health risk due to their epidemic potential and that they have no, or insufficient, countermeasures against them [23]. The WHO furthermore conducts an annual review of the Blueprint priority list [24]. Ebola, Marburg, Lassa, MERS-CoV, Nipah, and Rift Valley fever (RVF) viruses were among the viruses listed in 2018 [24]. That same year alone, six of the 10 priority pathogens listed in the WHO R&D Blueprint caused outbreaks [25]. “Disease X” is also listed: It represents the fact that a serious international epidemic could be caused by a pathogen currently unknown to cause human disease, toward which it is important to enable cross-cutting R&D preparedness [24].

CEPI is prioritizing investments in two areas. The first is the development of vaccines against a set of high-priority pathogens, which currently include Lassa, MERS-CoV, Nipah, RVF, and Chikungunya viruses. The second is the development of vaccine platform technologies that will enable rapid vaccine development and manufacturing to improve global capacity to respond to the emergence of an unknown pathogen with epidemic potential (Disease X) [26].

### Calls for proposals successfully announced

Since its launch, CEPI has announced three calls for proposals (CfP). The first and third CfP focused on CEPI’s priority pathogens, supporting candidate vaccines against MERS-CoV, Nipah, Lassa, RVF, and Chikungunya viruses. The second CfP aims to advance rapid-response platforms against unknown pathogens.

CEPI has established multiple partnering agreements that make up its current portfolio of 19 priority pathogen vaccine candidates and three rapid response platforms that reflect a potential investment of over US$450 million. Additional partnerships are under negotiation. Table 2 provides some details of the CEPI vaccine portfolio. These details are also provided on the CEPI website (www.cepi.net).

**Table 2:** The Coalition for Epidemic Preparedness Innovations (CEPI) funded projects (as of November 2019)

Partner	Disease	CEPI funding	Technology platform	Development phase
Janssen Vaccines and University of Oxford	Lassa	US$19.0 million	Recombinant virus	Preclinical
	MERS-CoV			Phase I
	Nipah			Preclinical
Profectus BioSciences, Emergent BioSolutions, and PATH	Lassa	US$36.0 million	Recombinant virus	Preclinical
	Nipah	US$25.0 million	Protein subunit	Preclinical
International AIDS Vaccine Initiative (IAVI)	Lassa	US$54.9 million	Recombinant virus	Preclinical
IDT Biologika	MERS-CoV	US$36.0 million	Recombinant virus	Phase I
Themis Bioscience	Lassa	US$58.5 million	Recombinant virus	Phase I
	MERS-CoV			Preclinical
	Chikungunya			Phase III
University of Tokyo	Nipah	US$31.0 million	Recombinant virus	Phase I
Inovio Pharmaceuticals	Lassa	US$56.0 million	DNA	Preclinical
	MERS-CoV			Phase II
Colorado State University	Rift Valley fever	US$9.5million	Attenuated virus	Preclinical
Wageningen Bioveterinary Research	Rift Valley fever	US$12.5 million	Attenuated virus	Phase I
Valneva	Chikungunya	US$23.4 million	Attenuated virus	Phase I
Public health vaccines	Nipah	US$43.6 million	Recombinant virus	Preclinical
Imperial College London	Marburg	US$8.4 million	RNA	Preclinical
	Influenza			
	Rabies			
CureVac	Lassa	US$34.0 million	RNA	Preclinical Phase I
	Rabies			
	Yellow fever			
University of Queensland	MERS-CoV	US$10.6 million	Recombinant protein	Preclinical
	Influenza			
	Respiratory syncytial virus			

The CEPI vaccine portfolio contains a wide variety of technologies, ranging across recombinant viral vectors, nucleic acid-based approaches, and recombinant proteins. Given that vaccine development is largely an empirical science, it is difficult to determine in advance which technology is likely to succeed in the clinic. Therefore, CEPI has invested in developing multiple candidates for each of its priority pathogens. For these priority pathogen projects, CEPI will seek to advance vaccine candidates through phase II clinical trials and the generation of an investigational stockpile. Such investigational stockpiles could be used during future outbreaks and in further clinical trials. For vaccine technologies enabling rapid response, CEPI’s initial investments will seek to demonstrate preclinical immunity to three pathogens and clinical (phase I) responses to two of these. In all cases, awards are made with stringent milestones and stage gates. The partnership arrangements that have been established also provide provisions ensuring that CEPI’s equitable-access goals can be achieved.

Currently, CEPI has invested in five technologies for Lassa fever vaccines using recombinant viruses and nucleic acid-based immunization. Indeed, the first CEPI-sponsored phase I clinical trial began in May 2019 using Inovio Pharmaceuticals’ DNA technology (NCT03805984) [27]. More recently, another phase I clinical trial for a Lassa vaccine candidate was initiated by Themis Bioscience (NCT04055454). There are four vaccine candidates under investigation for MERS-CoV, again with a similar combination of technologies. However, two of the recombinant viruses being tested have already gained useful clinical data prior to CEPI funding and thus are in a more advanced state of development. In the program for Nipah, four vaccines are in development and include recombinant viruses and recombinant proteins. In all cases, it is expected that phase I studies will begin within 18–24 months, with phase II studies following shortly afterward. Recent funding has been made available in partnership with the European Commission for RVF and Chikungunya. Two RVF vaccine candidates are in development and consist of attenuated viruses, other two candidates are under development for Chikungunya. Investments have also been made into three platform technologies based on nucleic acids and recombinant proteins. All three have the potential to produce a vaccine rapidly in the event of an emergency (Table 2). Additional investments in these areas will be announced shortly.

## Enabling science

International experts noted that for many of the diseases listed in the WHO R&D Blueprint, there is not only a need for a vaccine but also for developing a broader knowledge base of the disease itself. Basic and characterization research is needed, as well as epidemiological, entomological, and multidisciplinary studies; improved diagnostics; further elucidation of transmission routes; and social science research [24]. The knowledge built will be fundamental in the process of vaccine development.

To this purpose, CEPI has identified a set of research activities needed to accelerate vaccine development, and it is currently focusing on several enabling science projects related to the development of biological standards and assays, animal models, epidemiological studies, diagnostics, clinical trial capacity, and sustainable manufacturing. Although this list of research areas is not exhaustive, it represents a focused set of research activities and data collection priorities from a vaccine-development perspective.

### Biological standards and assays

Development of biological standards and assays is important for evaluating vaccine-elicited immune responses and promoting standardization, transparency, and comparability among the vaccine candidates. There are currently no available international antibody standards for Lassa, MERS-CoV, or Nipah, and there is a wide variety of intermediate standards currently used by Lassa vaccine developers.

CEPI, in collaboration with international partners, is collecting serum from patients from endemic countries who recovered from the actual diseases for the development of reference antibody preparations and, ultimately, Expert Committee on Biological Standardization (ECBS)-endorsed International Reference Preparations (IRPs). It is the aim of CEPI to make biological standards available to all CEPI-funded vaccine developers as early as possible, and for this purpose CEPI has established a Working Group on Standards, Assays and Animal Models, which is co-chaired by the WHO. In addition to this overarching group of experts, pathogen-specific task forces have also been established to obtain advice on specific topics related to standards, assays, and animal models. The task forces are instrumental in describing major needs for each disease, providing technical advice, and fostering collaboration across projects. These disease-specific task forces engage scientists from various geographic regions and from multiple disciplines. Moreover, CEPI also seeks to make pathogen-specific antigens available to relevant CEPI-funded vaccine developers. When moving toward phase I/II and, potentially, phase III efficacy trials, access to common sets of reference standards will be crucial for the evaluation of the vaccine and the comparison of different vaccine candidates.

As an example, in the past year CEPI launched requests for proposals and signed several partnership agreements for the distribution of Lassa virus-specific antigens and the development of a Lassa antibody standard. In collaboration with the Viral Hemorrhagic Fever Consortium (VHFC), the Bernhard Nocht Institute for Tropical Medicine (BNITM), and the National Institute of Biological Standards and Control (NIBSC), CEPI is collecting serum from individuals who recovered from the disease in endemic countries (Sierra Leone, Liberia, Mali, and Nigeria) for the development of reference antibody preparation and, ultimately, an IRP available to all globally.

### Animal models

Due to the nature of EIDs, obtaining human efficacy data may prove challenging for the vaccines in CEPI’s portfolio. Consequently, evidence of vaccine efficacy may need to rely, either in part or fully, on data from validated animal models acceptable to regulatory authorities. In 2002 the U.S. Food and Drug Administration (FDA) finalized the Animal Efficacy Rule (also known as the Animal Rule), which applies to the development and testing of drugs and biologicals to reduce or prevent serious and life-threatening conditions caused by exposure to lethal agents for which human efficacy trials are not feasible or ethical [28]. According to this rule, the FDA relies on animal studies to provide substantial evidence of product effectiveness akin to a traditional phase III clinical efficacy study. This route of licensure still requires human safety and immunogenicity, however. To rely on animal efficacy, much work needs to be done to build the foundation of data, such as natural history studies of one or more of the animal species selected, a reasonably well-understood mechanism for the toxicity of the pathogen, and pharmacokinetics and pharmacodynamics data sufficiently well understood to allow the selection of an effective dose in humans [29].

Therefore, CEPI is planning to support animal model development/refinement and natural history studies that can serve as a basis for qualification of the model by regulatory agencies. It is aligning with the National Centre for the Replacement, Refinement and Reduction of Animals in Research (NC3Rs) guidelines to accelerate the development of models and tools to avoid the use of animals where possible, reduce the number of animals used per experiment, minimize animal suffering, and improve welfare [30]. CEPI is currently mapping existing efforts and funding for such work and will explore collaborations and co-funding mechanisms as appropriate to avoid duplication of efforts in this space. The WHO has developed target product profiles (TPPs) for many of the priority pathogens, and CEPI uses the WHO TPPs as guiding documents to make many of its decisions regarding the feasibility and intended use of funded vaccines [31–33].

### Diagnostics

Diagnostic tests can serve multiple functions, including epidemiological surveillance, diagnosis in efficacy trials, case detection, and outbreak response. CEPI focuses on supporting the development of diagnostic tests to prepare for phase IIb/III clinical trials and identify cases of disease. Its efforts are in mapping the needs around the development of diagnostic tools, without which CEPI vaccine candidates cannot be advanced.

CEPI has limited funding for diagnostics-related activities; therefore, the diagnostic work is mainly accomplished through establishing partnerships and collaboration with potential product development partners. The Foundation for Innovative New Diagnostics (FIND) and CEPI have developed a partnership framework called CEPI.dx to address diagnostic needs for priority pathogens, and CEPI recently funded FIND with a total of US$1 million to support the evaluation of serological assays (IgG, IgM ELISA), clinical trial site development, and laboratory capacity strengthening in Lassa-affected countries. CEPI has also actively supported FIND’s application for the mobilization of a total of €4.2 million from the Federal Ministry for Education and Research (BMBF) of the German government. This funding has been used to support clinical evaluation of the Altona RealStar Lassa Virus RT-PCR Kit 2.0 (Altona Diagnostics, Hamburg, Germany), strengthening outbreak surveillance, research capacity, and activities related to biobanking in Lassa-affected countries.

### Epidemiological studies

Epidemiological studies are essential to understand the incidence and prevalence of EIDs, as well as their clinical characteristics and risk factors. These data are also essential to assess the feasibility of clinical field efficacy trials of promising vaccine candidates. Some CEPI-funded vaccine candidates have already entered testing in phase I clinical trials. If these initial trials are successful and vaccine candidates are deemed safe to proceed to the next stages of testing, further vaccine phase IIa trials in affected countries, and potentially phase IIb trials, will be conducted. To ensure the feasibility of efficacy trials and to support trial design, quality epidemiological data is needed. Epidemiological research can also help strengthen site and investigator capacity to conduct clinical trials. Therefore, CEPI is providing grants for epidemiological studies that aim to collect data that can contribute to vaccine development in support of trial design, appropriate end points, and site capacity.

To accelerate Lassa vaccine development, CEPI promoted an open call for research groups/consortia across Nigeria, Benin, Sierra Leone, Guinea, and Liberia to develop a core study protocol for a major multinational epidemiological study. This epidemiological study will be supported by an effort to develop and validate diagnostic assays in collaboration with FIND. Moreover, clinical trial site development and the establishment of one fully accredited clinical trial site and two to three sites in Nigeria, meeting Good Clinical Laboratory Practice (GCLP) standards for reliable diagnosis of Lassa fever cases, will be carried out to support future trials of vaccines. This will allow expanded sample collection and archiving to accelerate the research and development and regulatory approvals for new diagnostics and vaccines [23, 24].

### Building clinical trial capacity and exploring regulatory pathways

In addition, CEPI will provide support with respect to the clinical development of vaccine candidates, as well as in exploring regulatory pathways. The aim is to conduct clinical trials in affected endemic countries as early in the development as possible. CEPI will support the identification of clinical trial sites covering target populations and will engage in capacity building. It cooperates with the Brighton Collaboration, an international network of pharmacovigilance experts, to, among other activities, develop case definitions for potential adverse events of special interest (AESIs), including, for example, sensorineural hearing loss for the safety evaluation of Lassa vaccine candidates [34]. Moreover, the Brighton Collaboration will provide expertise in program-specific (upon request) and cross-program pharmacovigilance, for example by establishing a metadata safety monitoring board (meta-DSMB).

As CEPI’s priority pathogens mainly result in outbreaks, it is essential to explore the feasibility of field efficacy trials (phase IIb or III). CEPI will provide support in scenario planning, clinical trial design, capacity building, and so on for advanced-stage clinical trials to prove the vaccine candidate’s efficacy against infection, disease, or both. These advanced-stage clinical trials will have to be placed in an overall clinical development plan that is aligned and supported by the relevant regulatory authorities, as well as the WHO prequalification group. In this context, CEPI will also explore alternative regulatory pathways in case vaccine efficacy cannot be demonstrated in field trials, for instance when there is rapid decline of the infectious disease outbreak or no ongoing outbreak.

### Sustainable manufacturing

After the generation of an investigational stockpile for the candidate vaccines, a sustainable supply of vaccine will be critical. Cost and time efficiency during manufacturing for future stockpiles, outbreak response, and routine use of new vaccines in endemic regions will be of great importance. Since many of the vaccines CEPI is developing will not find commercial markets to sustain them, CEPI is exploring different approaches to provide for the long-term manufacturing of any successful vaccine candidates and is considering establishing advanced manufacturing partnerships with a limited number of public and private-sector manufacturing organizations. Ongoing efforts to understand potential epidemic scenarios and to model supply chain and stockpile requirements will contribute to this effort.

## Conclusions

Vaccines are a powerful tool with substantial potential to prevent and control outbreaks of EIDs. However, a key issue is to be able to manufacture and test a safe and efficacious vaccine for the immediate threat within a very short time frame. Prior to CEPI’s establishment, vaccine development efforts for EIDs were fragmented, with no sustainable mechanism to support successful projects across the vaccine development life cycle nor to coordinate work toward the highest-priority global epidemic risks. CEPI was created to fill these gaps and to stimulate, finance, and coordinate the development of vaccines against EIDs, especially in cases where market incentives alone were failing to drive needed development.

From its creation, CEPI developed a business plan to advance vaccine candidates to the PoC stage (by supporting phase I and II clinical trials) to enable clinical efficacy testing (phase III) during outbreaks. It is also CEPI’s intent to contribute to technical and institutional platforms that can accelerate the R&D response to EIDs. CEPI will continue to coordinate closely with the WHO and to work together with the international community to advance vaccine candidates against EIDs with epidemic potential, to establish and maintain investigational stockpiles of promising candidates, and to implement scientifically robust trials of these candidates during outbreaks.

## Abbreviations

AESIs: Adverse events of special interest
BARDA: Biomedical Advanced Research and Development Authority
BMBF: Federal Ministry for Education and Research
BMGF: Bill & Melinda Gates Foundation
BNITM: Bernhard Nocht Institute for Tropical Medicine
CDC: Centers for Disease Control and Prevention
CEPI: Coalition for Epidemic Preparedness Innovations
CMC: Chemistry, Manufacturing and Controls
DRC: Democratic Republic of the Congo
DS/DP: Downstream/upstream
DTRA: Defense Threat Reduction Agency
EC: European Commission
ECBS: Expert Committee on Biological Standardization
EIDs: Emerging infectious diseases
GAVI: Global Alliance for Vaccines and Immunization
GCLP: Good clinical laboratory practice
GDP: Gross domestic product
GloPID‑R: Global Research Collaboration for Infectious Disease Preparedness
GMP: Good manufacturing practice
IAVI: International AIDS Vaccine Initiative
IMI: Innovative Medicines Initiative
IRPs: International reference preparations
LMIC: Low-income and middle-income countries
MERS-CoV: Middle East respiratory syndrome coronavirus
Meta-DSMB: Metadata safety monitoring board
MSF: Médecins Sans Frontières
NC3Rs: National Centre for the Replacement, Refinement and Reduction of Animals in Research
NIBSC: National Institute for Biological Standards and Control
NIH: National Institutes of Health
PAHO: Pan American Health Organization
PDs: Product developers
PoC: Proof of concept
R&D: Research and development
RVF: Rift Valley fever
SAC: Scientific advisory committee
SARS-CoV: Severe acute respiratory syndrome-related coronavirus
SNHL: Sensorineural hearing loss
SOPs: Standard operating procedures
TPPs: Target product profiles
UNICEF: United Nations International Children’s Emergency Fund
VHFC: Viral Hemorrhagic Fever Consortium
VLPs: Virus-like particles
WHO: World Health Organization
